# Benchmark Study of Core-Ionization Energies with the Generalized Active Space-Driven Similarity Renormalization Group

**DOI:** 10.1021/acs.jctc.4c00835

**Published:** 2024-09-13

**Authors:** Meng Huang, Francesco A. Evangelista

**Affiliations:** Department of Chemistry and Cherry Emerson Center for Scientific Computation, Emory University, Atlanta, Georgia 30322, United States

## Abstract

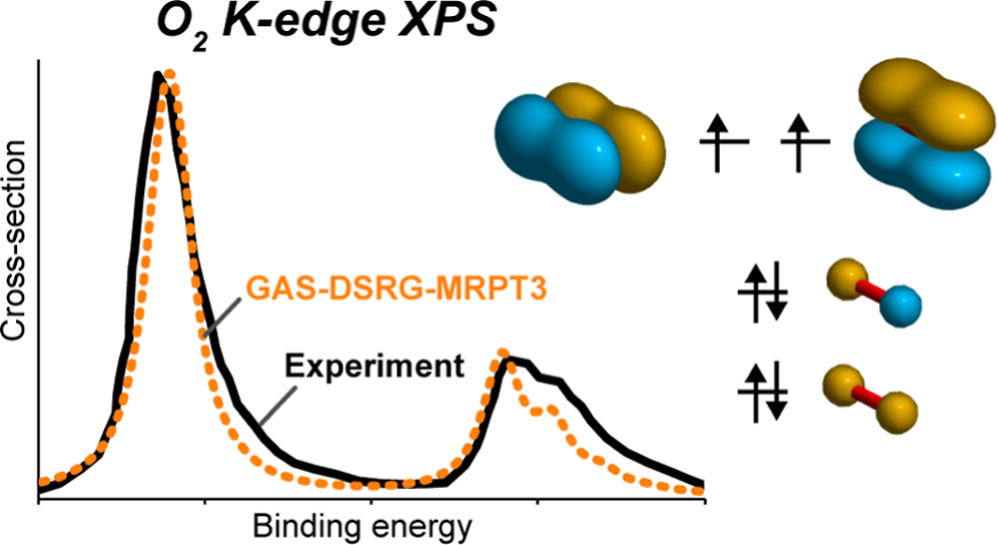

X-ray photoelectron spectroscopy (XPS) is a powerful experimental technique for probing the electronic structure of molecules and materials; however, interpreting XPS data requires accurate computational methods to model core-ionized states. This work proposes and benchmarks a new approach based on the generalized active space-driven similarity renormalization group (GAS-DSRG) for calculating core-ionization energies and treating correlation effects at the perturbative and nonperturbative levels. We tested the GAS-DSRG across three data sets. First, the vertical core-ionization energies of small molecules containing first-row elements are evaluated. GAS-DSRG achieves mean absolute errors below 0.3 eV, which is comparable to high-level coupled cluster methods. Next, the accuracy of GAS-DSRG is evaluated for larger organic molecules using the CORE65 data set, with the DSRG-MRPT3 level yielding a mean absolute error of only 0.34 eV for 65 core-ionization transitions. Insights are provided into the treatment of static and dynamic correlation, the importance of high-order perturbation theory, and notable differences from density functional theory in the predicted energy ordering of core-ionized states for specific molecules. Finally, vibrationally resolved XPS spectra of diatomic molecules (CO, N_2_, and O_2_) are simulated, showing excellent agreement with experimental data.

## 1 Introduction

X-ray photoelectron spectroscopy (XPS) has become a cornerstone spectroscopic technique, particularly in materials science and chemistry,^1^ since it was first demonstrated in 1957.^2^ In an XPS experiment, X-ray radiation ionizes a core electron from the sample, and the kinetic energy of the ionized electron is then analyzed.^3^ One of the advantages of XPS is that the ionization process is always allowed, enabling the identification of both bright and dark core-excited states. Though most XPS studies are focused on investigating local electronic properties and the chemical composition of materials, the emergence of time-resolved XPS (TR-XPS) techniques^4−10^ has enabled the extension of XPS to track microscopic dynamic processes that occur in chemical and photochemical reactions.

Interpreting data produced in new X-ray-based experiments requires accurate and cost-effective electronic structure calculations for the core-excited and core-ionized states. Many computational approaches have been developed for describing core-excited states, which can be roughly divided into two categories: efficient density functional theories^11−34^ and costly but systematically improvable wave function-based methods.^8,35−80^ Several electronic structure methods have been developed to simulate core-ionization states from closed-shell ground-state wave functions, such as the equation-of-motion ionization potential coupled cluster (EOMIP-CC)^8,45,51,60,61,65,66,75^ and the algebraic diagrammatic construction (ADC).^81−83^ However, these methods may encounter challenges in describing simple diatomic systems such as CO, where the treatment of static correlation for the near-degenerate π orbitals leads to different binding energies,^84^ and the higher core-excited states of CO^+^ show significant spin coupling effects.^85^ These challenges can be addressed by incorporating higher-order excitations in single-reference approaches, such as coupled-cluster methods,^55,86^ though this comes with a significant increase in computational cost. As a result, various multireference approaches^69,75,83^ have been developed for core-ionized states with the intention of overcoming the above-mentioned limitations and improving the accuracy of potential energy surfaces, especially those far from the equilibrium molecular structure.

We have recently developed a new multireference generalized active space-driven similarity renormalization group (GAS-DSRG) scheme that can accurately model core-excited states. This approach is based on general active space self-consistent field (GASSCF) reference wave functions combined with a driven similarity renormalization group (DSRG) treatment of dynamical electron correlation effects.^87^ Its accuracy in simulating XAS of small to large molecules has been benchmarked against experimental results and EOM-CC methods.^88^ In contrast to EOM methods, the GAS-DSRG is best suited to compute a small number of states; however, the GAS-DSRG yields highly accurate core-excitation energies since it can directly account for orbital relaxation (hybridization) effects and multielectron excited states.

In this study, we aim to extend the GAS-DSRG to model core-ionization transitions relevant to XPS and TR-XPS. To this end, we have performed a comprehensive benchmark for various systems, simulating vertical transition energies and spectra built from potential energy surfaces computed with the GAS-DSRG approach. The article is organized as follows. First, in Section 2, we provide an overview of the GAS-DSRG approach. Next, in Section 3, we outline the computational procedures employed for all benchmark studies. In Section 4, we present the results of our benchmark studies for core-ionized states. The first set of results considers core-ionization energies of small molecules from the benchmark set of Liu et al.,^65^ enabling a comparison between GAS-DSRG, coupled cluster, and MR-ADC^83^ methods. The second set of results tests the GAS-DSRG approach on core-ionized states of relatively large organic molecules using the CORE65 set of Golze et al.^31^ The final set of results focuses on the simulation of vibrationally resolved XPS of diatomic molecules, which are then compared with experimental and theoretical calculations such as ADC(4).^89−91^ Finally, in Section 5, we summarize our findings and provide insights into future applications of the GAS-DSRG scheme.

## 2 Theory

In this section, we give a brief summary of the GAS-DSRG approach introduced in our previous studies on core-excited states,^87,88,92^ which is based on the multireference DSRG formalism discussed in refs (93 and 94).

In the GAS-DSRG scheme, a set of GASSCF zeroth-order reference states are used to model the ground state (Ψ_0_) and core-ionized states (Ψ_α_, α = 1, 2, ..., *m*). These states are represented as follows
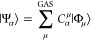
1where |Φ_μ_⟩ is a determinant that satisfies the GAS restrictions and *C*_α_^μ^ is the corresponding coefficient. In the GAS approach, the orbitals are divided into core (doubly occupied), virtual (empty), and a variable number of active subspaces (GAS*n*, where *n* = 1, 2, ...). By setting limits on the number of electrons allowed to occupy each GAS*n* space, it is possible to target electronic states characterized by a specific occupation pattern. Specifically, for core-ionized states, the GAS1 space includes core orbitals from which an electron is removed. The GAS2 space comprises specific occupied and unoccupied valence orbitals required to correctly model the ground state, e.g., along a bond-breaking reaction coordinate.

The ground- and core-ionized states are built from distinct sets of orbitals, with the latter optimized in a state-specific or state-averaged way. The separate treatment of orbital optimization is an important aspect of the GAS-DSRG approach and allows the method to accurately capture relaxation effects following core-ionization. In the GASSCF computations for core-ionized states, we freeze the number of electrons within each GAS and exclude orbital rotations between different GAS subspaces. This is done to prevent collapse of the core-ionized state into the ground state of the electron-detached molecule. For most small molecules, we target the dominant core-ionized state with a state-specific formalism. We employ a state-averaged formalism instead for molecules with near-degenerate core orbitals (ethane, trifluoromethane, acetone, acetic acid, nitrobenzene, benzene, and phenylacetylene). Note that our current GAS approach for core-ionized states can be equivalently implemented using restricted active space (RAS)^95,96^ and occupation restricted multiple active space (ORMAS)^97^ approaches.

After generating the GASSCF reference states, we include the missing dynamical electron correlation via the multireference DSRG (MR-DSRG) approach.^94^ In the MR-DSRG theory, we build an effective Hamiltonian via the unitary transformation
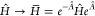
2where the operator  is anti-Hermitian and expressed in terms of a generalized form of the coupled cluster excitation operator . This unitary transformation removes the so-called nondiagonal part, , which corresponds to the components of  that couple the GAS determinants to determinants outside this space. In principle, once this decoupling is achieved [i.e., ], we can obtain the exact eigenvalues for a manifold of states by diagonalizing  in the space of GAS determinants.

To prevent numerical instabilities that arise from small denominators (which are associated with the intruder-state problem) when solving for the condition , in the DSRG, we introduce a term that regularizes the equation and depends on the *flow parameter* (*s*)
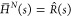
3Here, the off-diagonal terms  are gradually driven to zero by the *source* operator, . As *s* → *∞*, we recover the condition . The flow parameter controls the magnitude of the amplitudes in the  operator, preventing excitations with small denominators from diverging. The choice of the flow parameter affects the accuracy of MR-DSRG results, and optimal ranges have been identified through benchmarking of ground states^98^ and core-excitation energies.^87,88^ Once the DSRG equation [eq 3] is solved, the energy is obtained by diagonalizing the DSRG Hamiltonian  in the space of GAS determinants
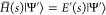
4where *E*′(*s*) and |Ψ′⟩ are the *relaxed* energy and reference, respectively.^93,99−103^ Note that in the state-averaged variant, we solve for multiple solutions of the eigenproblem given in eq 4.

In this work, we performed most of the core-ionized state calculations using perturbative approximations of the MR-DSRG up to second and third-order (DSRG-MRPT*n*, *n* = 2, 3).^93,101^ In these treatments, the zeroth-order Hamiltonian is the diagonal component of the generalized Fock matrix, assuming a semicanonical basis. The availability of efficient implementations of these DSRG perturbative methods^103^ (which require at most the three-body reduced density matrices of the GAS references) allows us to quickly perform DSRG-MRPT2/3 calculations on relatively large molecules with multiple targeted core atoms. For the calculations on the core-ionization energies of small molecules, we also consider a nonperturbative approximation to the DSRG termed MR-LDSRG(2), which truncates *Â* to one- and two-body substitution operators and approximates the effective Hamiltonian by neglecting three- and higher body terms.^100^

## 3 Computational Details

To evaluate the core-ionization energies with the GAS-DSRG approach, we start by determining mean-field level guess orbitals, with the exception of linear or highly symmetric molecules with degenerate ground states, for which we use state-averaged CASSCF to retain the symmetry of the initial electron-detached state. Next, we optimize each set of orbitals using GASSCF, with the core orbital(s) kept frozen. Finally, we perform separate DSRG calculations to obtain the GAS-DSRG energy of the ground and core-ionized states, and the ionization energy is obtained as the difference between the two. All the calculations are performed using the forte(104) package with integrals obtained from Psi4.^105^

We comprehensively benchmarked the accuracy of GAS-DSRG theories on core-ionization energies using three different test sets. In the first set, we evaluated the core-ionization energies of small molecules containing one or two first-row elements to test our method’s accuracy compared to existing highly accurate wave function methods such as core–valence separated (CVS) EOM-CC (CVS-EOM-CC)^65^ and ΔCC.^66^ The vertical transition energies are evaluated using the DSRG-MRPT2, DSRG-MRPT3, and MR-LDSRG(2) levels of theory, the cc-pCVXZ-DK (X = T, Q) basis sets, and scalar X2C relativistic corrections.^106−110^ We used the molecular geometries from the study of Liu et al.,^65^ where diatomic molecular geometries are taken from experiments, and the geometries of polyatomic molecules are optimized at the SFX2C-1e-CCSD(T)/cc-pCVQZ^107,109^ level of theory. We used a full-valence active space for all of the molecules in this set. For the DSRG-MRPT2/3 computations, we chose *s* = 1*E*_h_^–2^, an optimal value for core-excitation energies for perturbative approximations,^88^ while we use *s* = 0.5*E*_h_^–2^ for the highly accurate MR-LDSRG(2) theories as it leads to improved convergence.

In the second set, we tested the ability of our GAS-DSRG theories to accommodate relatively large organic systems by utilizing the CORE65 set,^31^ which contains 65 binding energies from various gas-phase molecules of sizes up to 14 atoms. Similar to our previous calculations for small molecules, these binding energies correspond to vertical core-ionization energies. We calculated the core-ionization energies for first-row elements using the DSRG-MRPT2/3 levels of theory and the cc-pVQZ basis set without including relativistic corrections. The absence of these corrections results in an estimated shift of 0.2 eV in the transition energy. The geometries from the CORE65 set are optimized at the density functional theory (DFT) level using numeric atom-centered orbitals of tier 2 quality.^111^ For larger systems with multiple ionization channels, we applied the state-averaged DSRG formalism.^88,94^ The detailed choice of active space and the number of states considered for each molecule are listed in the Supporting Information (Table S1). The default flow parameter value for these calculations is *s* = 1 *E*_h_^–2^, except in cases in which the root-flipping issue causes the DSRG-MRPT2 level of theory to fail.

Finally, we simulated vibrationally resolved XPS bands in CO,^89^ N_2_,^91^ and O_2_^90^ to test how well our method performs on core-ionized states far from the equilibrium region. Specifically, we simulated the vibrational structure of XPS using the Franck–Condon factors generated from potential energy scans with an interval of 0.05 Å, evaluated with GAS-DSRG-MRPT2/3 theories and cc-pCVQZ-DK basis sets.^112−114^ We used full valence and core orbitals as the active space for GAS calculations. For the *s* value in these DSRG calculations, we still use *s* = 1 *E*_h_^–2^. Scalar X2C relativistic effects are also included. We obtained the vibrational eigenvalues and eigenvectors using the discrete variable representation (DVR) method,^115,116^ with the potential energy values at each grid point evaluated through cubic spline interpolation.

## 4 Results and Discussion

### 4.1 Vertical Core-Ionization Energy of Small Molecules

We first evaluated the accuracy of GAS-DSRG on K-edge ionization energies of first-row elements using a test set of molecules containing no more than three non-hydrogen atoms. Table 1 presents the error of three different levels of DSRG theories [DSRG-MRPT2, DSRG-MRPT3, and MR-LDSRG(2)] compared to experimental results. All GAS-DSRG theories agree with experiment, with a mean absolute error (MAE) of less than 0.3 eV and a standard deviation (STD) of less than 0.2 eV. These small MAEs are comparable to the results from the EOMIP-CCSDT level of theory (MAE = 0.14 eV) under the same basis,^65^ especially for the DSRG-MRPT2 level of theory (MAE = 0.16 eV). Compared to the MR-ADC(2)-X approach (MAE = 0.46 eV) based on a similar multideterminantal many-body expansion, our GAS-DSRG theories exhibit a smaller error. This improvement can be attributed to the optimization of orbitals for the core-ionized states in the presence of static correlation. The importance of orbital optimization is also evident from the small error at the ΔCCSD/ΔCCSD(T) levels of theory using the cc-pVTZ basis (MAE = 0.17/0.18 eV),^66^ which use different orbitals for ground and core-ionized states.

**Table 1:** Experimental K-Edge Core-Ionization Energies of First-Row Elements for Small Molecules and the Errors from GAS-DSRG Results (in eV)^a^

	Exp	error (PT2)		error (PT3)		error [LDSRG(2)]	
		TZ	QZ	TZ	QZ	TZ	QZ
H**F**	694.23	0.27	0.43	0.26	0.32	0.28	0.38
**C**O	296.21	–0.01	0.01	0.04	0.05	0.10	0.09
C**O**	542.55	0.27	0.14	0.27	0.19	0.26	0.28
**N**_2_	409.98	–0.01	–0.02	0.21	0.25	0.21	0.24
**F**_2_	696.69	–0.26	–0.19	0.63	0.72	0.45	0.56
H_2_**O**	539.90	0.04	0.15	0.09	0.10	0.14	0.19
**C**_2_H_4_	290.82	0.01	–0.14	0.38	0.31	0.35	0.24
**C**_2_H_2_	291.14	0.14	–0.01	0.49	0.45	0.48	0.37
**C**H_4_	290.91	0.13	0.03	0.23	0.10	0.25	0.12
CH_2_**O**	539.48	0.38	0.28	–0.03	0.02	0.34	0.39
**C**H_2_O	294.47	0.16	0.12	0.16	0.11	0.18	0.13
C**O**_2_	541.28	0.11	0.16	0.52	0.53	0.39	0.44
**C**O_2_	297.69	0.24	0.21	0.09	0.09	0.17	0.16
NN**O**	541.42	0.25	0.32	0.19	0.24	0.24	0.28
N**N**O	412.59	0.27	0.28	0.25	0.26	0.28	0.28
**N**NO	408.71	0.05	0.09	0.06	0.10	0.09	0.12
**N**H_3_	405.56	0.02	0.07	0.12	0.10	0.27	0.25
HC**N**	406.78	0.30	0.17	0.26	0.14	0.33	0.27
H**C**N	293.40	0.11	0.04	0.19	0.11	0.19	0.11
CH_3_**O**H	539.11	0.08	0.31	–0.08	0.33	0.37	0.35
**C**H_3_OH	292.43	0.14	0.14	0.36	0.35	0.30	0.19
MAE		0.15	0.16	0.23	0.23	0.27	0.26
STD		0.14	0.15	0.18	0.18	0.11	0.12

a GAS-DSRG results were computed with a GASSCF reference and different levels of theories [PT2 = DSRG-MRPT2, PT3 = DSRG-MRPT3, and LDSRG(2) = MR-LDSRG(2)] using the cc-pCVTZ-DK/cc-pCVQZ-DK basis and a scalar X2C relativistic correction. The bold element denotes the atom from which the core electron is ionized. We also reported statistics for the GAS-DSRG results (MAE = mean absolute error; STD = standard deviation).

Our evaluation of different basis sets and GAS-DSRG theories revealed interesting findings. The difference in STD and MAE between cc-pCVTZ-DK and cc-pCVQZ-DK for all GAS-DSRG theories is less than 0.01 eV, indicating that the GAS-DSRG core-ionization energies are nearly converged with respect to the basis sets. However, the results show a more significant dependence on the truncation level of GAS-DSRG theories. The DSRG-MRPT2 level of theory shows the best agreement with the experimental values (MAE = 0.16 eV), while the DSRG-MRPT3 (MAE = 0.23 eV) and MR-LDSRG(2) (MAE = 0.26 eV) results consistently show larger deviations, likely due to error cancellation. Interestingly, for the same benchmark set, the highly accurate ΔCC method suffers from larger errors (∼0.5 eV MAE) under the same cc-pCVTZ and cc-pCVQZ basis sets.^66^ This deviation was found to be caused by the core–valence separation and could be reduced by adding a CVS correction. In contrast, the DSRG-MRPT3 and MR-LDSRG(2) results obtained with the same basis show smaller deviations (∼0.26 eV) even though our GAS treatment fixes the number of electrons in the orbitals from which the electron is ionized. We are currently studying to what extent the restrictions imposed in the GAS-DSRG approach correspond to the effects of the CVS observed in CC methods and their magnitude.

This first test set shows that there is excellent agreement between all of the GAS-DSRG levels of theory and experimental K-edge core-ionization energies on small molecules. In particular, these results show that the DSRG-MRPT3 method represents a good compromise between accuracy and computational efficiency in the evaluation of core-ionized states. As a result, in our next test cases, we focus only on the DSRG-MRPT2/3 level of theories, excluding the more expensive MR-LDSRG(2) computations.

### 4.2 CORE65 Set

**Figure 1:**
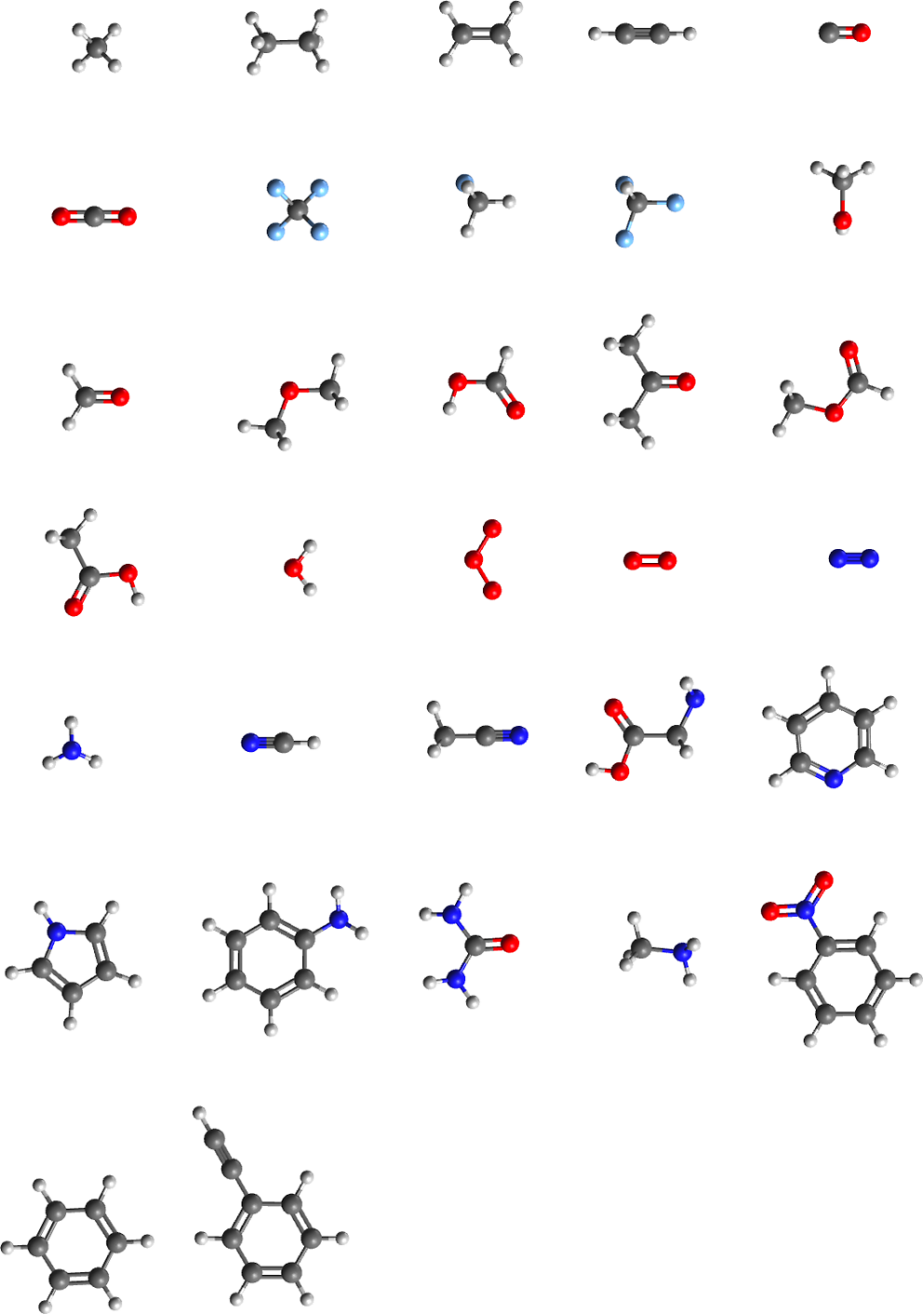
Structure of the molecules contained in the CORE65 benchmark set. Elements are color coded as follows: hydrogen (white), carbon (gray), nitrogen (blue), oxygen (red), and fluorine (light blue).

**Figure 2:**
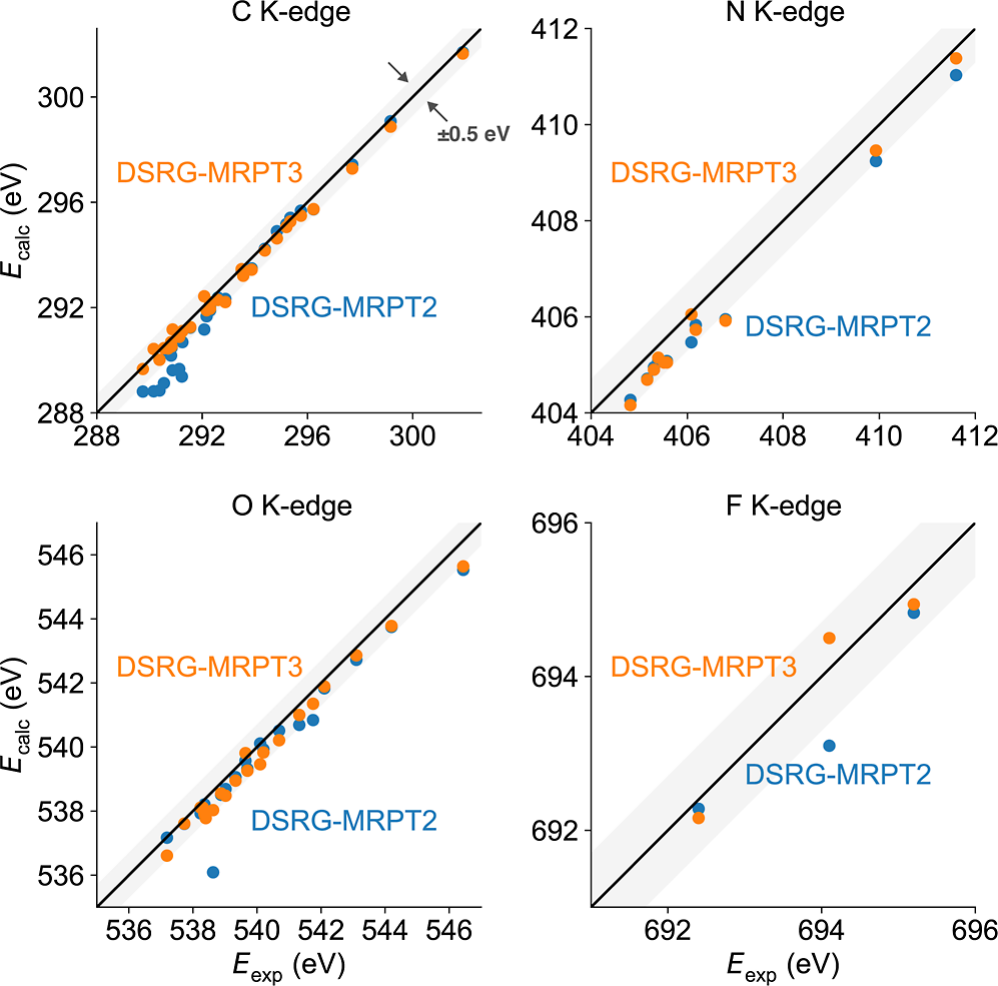
Comparison between theoretical and experimental C, N, O, and F K-edge transition energies in the CORE65 set. The DSRG transition energies are computed using DSRG-MRPT2 and DSRG-MRPT3 theories with the cc-pVQZ basis. For each scatter point, the horizontal and vertical axes indicate its experimental and theoretical values, with the diagonal line noting perfect agreement. The shaded area indicates the region where the calculated values deviate ±0.5 eV from the experimental values.

Next, we use the CORE65 data set^31^ to benchmark core-ionization energies computed with the GAS-DSRG approach for medium-size molecules. This set contains 32 inorganic and organic molecules with up to 14 atoms, as shown in Figure 1. Correlation plots between the experimental and DSRG-MRPT2/3 core-ionization energies (C, N, O, and F K-edge) for the CORE65 set are shown in Figure 2. Most DSRG-MRPT2 and nearly all DSRG-MRPT3 predictions are within 0.5 eV of the experimental core-ionization energies. The detailed transition energies are listed in the Supporting Information (Table S2). Table 2 illustrates the error statistics for the DSRG-MRPT2/3 levels of theory in evaluating the C, N, O, and F K-edge core-ionization energies compared to experiment. The overall MAE of the DSRG-MRPT3 theory is only 0.34 eV, making it accurate enough to interpret most XPS spectra. This value is comparable to the error from the more efficient evGW_0_ and G_0_W_0_ approaches using PBEh (α = 0.45) orbitals and the same basis set (0.89 and 0.58 eV, respectively). However, the GW errors decrease somewhat when extrapolated to the complete basis set (CBS) limit (0.30 and 0.33 eV).^31^ While Slater’s transition method (STM)^34^ can yield an even smaller MAE (<0.20 eV) compared to that of the experiment, the results can significantly depend on the exchange–correlation functional applied. The DSRG-MRPT3 results are consistent across all molecules, with the standard deviation being only 0.25 eV. The influence of the element type on the MAE is relatively small, with the C K-edge core-ionization energy MAE (0.26 eV) being smaller than the N/O/F K-edge ones (0.44/0.40/0.30 eV). This error distribution across different elements is comparable to that from the evGW_0_ theory (0.27/0.30/0.32/0.44 eV for C/N/O/F). More importantly, the error distribution of core-ionization energies is consistent with our previous benchmark of core-excited energies^88^ using the XABOOM set,^117^ where the DSRG-MRPT3 theory leads to a 0.32 eV MAE (0.27/0.37/0.38 eV for C/N/O K-edges) compared to available experimental values.

**Table 2:** Error Statistics (in eV; MAE = Mean Absolute Error; STD = Standard Deviation) of GAS-DSRG Theories on the CORE65 Data Set Compared to the Experiment^a^

		PT2		PT3	
	*N*_T_	MAE	STD	MAE	STD
all	65	0.50	0.48	0.34	0.25
C 1s	30	0.57	0.53	0.26	0.22
N 1s	11	0.52	0.16	0.44	0.23
O 1s	21	0.44	0.54	0.40	0.22
F 1s	3	0.50	0.46	0.30	0.38

a For each element, we list the number of transitions (*N*_T_). GAS-DSRG results were computed with a GASSCF reference and different levels of theory [PT2 = DSRG-MRPT2 and PT3 = DSRG-MRPT3] using the cc-pVQZ basis.

In contrast, the DSRG-MRPT2 level of theory shows lower accuracy and consistency, reflected by a notably higher MAE/STD of 0.50/0.48 eV. This is primarily attributed to significant errors at several outlier points. For example, the molecules with the largest experimental deviations are nitrobenzene (O 1s), acetone (C 1s in CH_3_), and benzene (C 1s), with errors of −2.54, −1.85, and −1.53 eV, respectively. The DSRG-MRPT2 theory’s deficiency for these transitions is mainly due to the intruder-state problem. This conclusion is supported by calculations on these outliers using *s* = 0.5*E*_h_^–2^, which result in smaller errors (−1.67, −1.30, and −0.93 eV, respectively). We made similar observations in previous benchmark results on the core-excited states of glyoxylic acid,^88^ but intruder states occur more frequently in computations of core-ionized states. Additionally, we already applied the small *s* = 0.5 *E*_h_^–2^ parameter when evaluating the core-ionized states of CH_3_F (F 1s) and nitrobenzene (C 1s) using DSRG-MRPT2 to avoid root-flipping issues. Although the DSRG-MRPT2 theory, when combined with a relatively small *s* value, can potentially serve as a reasonably accurate and consistent method for interpreting XPS experiments, DSRG-MRPT3 is still preferable due to its robustness and accuracy, especially for larger molecules.

**Figure 3:**
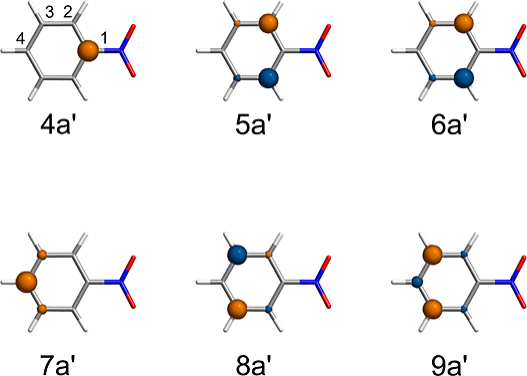
C 1s core orbitals of nitrobenzene used for GAS-DSRG calculations and atom numbering used in the paper. The orbitals are ordered in ascending order of their Hartree–Fock energies.

**Figure 4:**
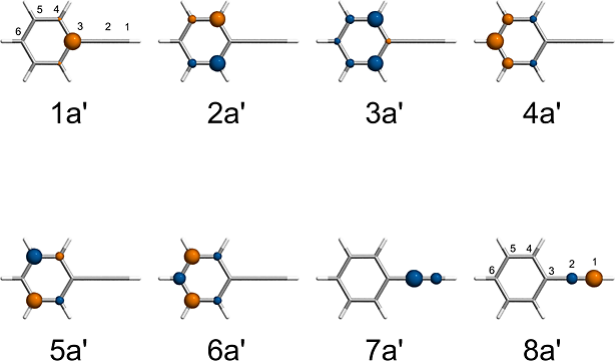
C 1s core orbitals of phenylacetylene used for GAS-DSRG calculations and atom numbering used in the paper. The orbitals are ordered in ascending order of their Hartree–Fock energies.

In the CORE65 set, we observed an interesting discrepancy among theories of the prediction of the core-ionized states of nitrobenzene and phenylacetylene. Both systems have multiple C 1s ionization channels, leading to multiple transitions observed in the XPS experiments. Our analysis using DSRG-MRPT3 has revealed that the lowest five core-ionized states in nitrobenzene are very closely spaced in energy, within 0.3 eV of each other. These states correspond to the ionization of one electron from the 4*a*′, 5*a*′, 6*a*′, 8*a*′, and 9*a*′ orbitals (C_1–3_), as shown in Figure 3. The sixth state, which is 1.54 eV higher than the lowest-core-ionized state, corresponds to the ionization of an electron from the 7*a*′ state (C_4_). This energy order contrasts with GW and Δ*S*CF calculations,^31,33^ where a much higher energy is assigned to the ionization of an electron from the C_1_ atom. It is worth noting that the order predicted by DSRG theories differs from the prediction by generalized Koopman’s theorem,^118^ where the ionization energy of an electron should be roughly equal to its HF orbital energy. Similar observations are found in the core-ionized states of phenylacetylene, which are even more complex. Figure 4 shows the core orbitals of phenylacetylene. According to DSRG-MRPT3, the first five core-ionized states in phenylacetylene arise from the ionization of electrons from the 2*a*′ to 6*a*′ orbitals, which are closely spaced within 0.2 eV of each other. The core-ionization state of the 8*a*′ electron (mainly C_1_) is very close to the core-ionization state of the 1*a*′ electron (mainly C_3_), which is lower than the highest core-ionization state of the 7*a*′ electron (mainly C_2_) by about 0.7 eV. This energy order of core-ionized states (C_4–6_ < C_1_ < C_3_ < C_2_) again differs significantly from the order predicted by GW calculations^31^ and a Δ*S*CF study^119^ (C_1_ < C_4–6_ < C_2_ < C_3_), while a more recent STM study suggests another order (C_4–6_ < C_2_ < C_1_ < C_3_). While the current experimental data do not provide a definitive conclusion for the energetic order, it is worth noting that two critical differences exist between the DSRG and GW results. First, the DSRG calculations rely on a state-averaged formalism that treats all core-ionized states on equal footing, while GW/Δ*S*CF calculations use a state-specific formalism. More importantly, DSRG is based on delocalized semicanonical molecular core orbitals, while previous Δ*S*CF^119^ and GW calculations^31^ are based on localized core orbitals. Higher-level computations on these systems could illuminate the debate surrounding the best way to treat core vacancies (either localized or delocalized) in XPS simulations.^120,121^

### 4.3 Vibrationally Resolved XPS of Diatomic Molecules

**Figure 5:**
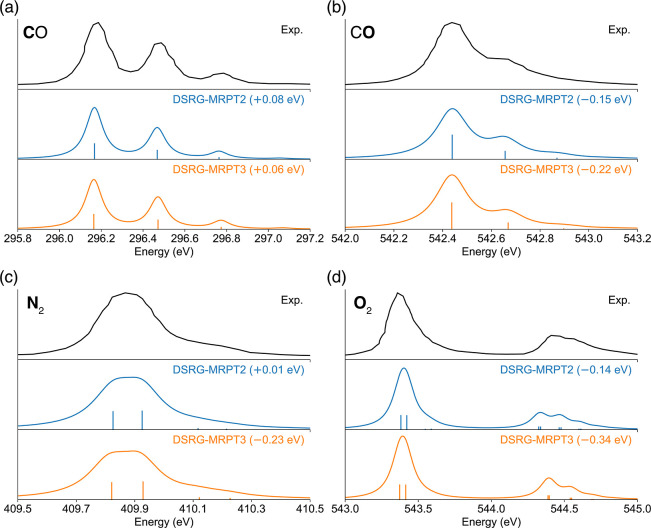
Vibrationally resolved XPS of (a) CO C 1s, (b) CO O 1s, (c) N_2_ N 1s, and (d) O_2_ O 1s, obtained from theoretical simulations using the DSRG-MRPT2 and DSRG-MRPT3 levels of theory and the cc-pCVQZ-DK basis set. The experimental spectra are digitized from refs (89–91). For best comparison, the simulated spectra have been shifted and labeled with their respective shifts in each trace. The Cohen–Fano coefficients for N_2_ are not included.^91^ To reproduce the experimental XPS of O_2_, we scale the doublet/quartet transitions to match the intensity ratio (2.20) observed experimentally.^90^

The previous two benchmark tests have shown that the GAS-DSRG approach yields accurate vertical core-ionization energies for molecules. However, one of the reasons for developing multireference approaches such as the GAS-DSRG is to compute XPS for transient molecules and systems away from their equilibrium geometry. To investigate the accuracy of the GAS-DSRG in these more challenging cases, we simulated the experimental vibrationally resolved XPS spectra of CO (C 1s and O 1s),^89^ N_2_ (N 1s),^91^ and O_2_ (O 1s).^90^Figure 5 shows both the experimental and simulated spectra for the four molecules. Both DSRG-MRPT2 and DSRG-MRPT3 theories lead to excellent agreement with the experimental XPS vibrational structure. The simulated spectra shifts required to match the experimental spectra, which represent the error in the zero-point corrected adiabatic transition energy compared to the experiment, are only 0.01–0.15 eV for DSRG-MRPT2 and 0.06–0.34 eV for DSRG-MRPT3. We also observed a slightly larger error from DSRG-MRPT3 theory compared to that from DSRG-MRPT2 in our previous simulation of vibrationally resolved XAS with the same level of theory and basis set, attributed to possible better error cancellation at the PT2 level.^87^ On the other hand, DSRG-MRPT3 leads to more accurate splittings between transitions. This is especially true for O_2_, where two peaks are observed in the experimental spectrum, corresponding to the ^4^Σ and ^2^Σ states of core-ionized O_2_^+^. The DSRG-MRPT3 theory predicts a splitting of 1.00 eV, which better agrees with the experimental value of 1.04 eV than the value predicted by DSRG-MRPT2 (0.92 eV).

We further explored the accuracy of potential energy curves by fitting them to Morse potentials and obtained the resulting vibrational constants. Although the cross-sections for core-ionization can be derived from multireference Dyson orbitals,^122^ we here approximated these cross-sections using Franck–Condon factors.^123^ Both the vibrational constants and the approximated cross-sections are reported in the Supporting Information (Tables S3 and S4). The DSRG-MRPT2/3 vibrational frequencies are all within 50 cm^–1^ from the experimental results and previous theoretical calculations, such as ADC(4)^82^ and symmetry-adapted cluster configuration interaction (SAC–CI).^124^ However, it is essential to note that the accuracy of these constants can be highly dependent on the range of potential energy curves used for fitting. The calculated Franck–Condon factors from DSRG-MRPT3 calculations are in excellent agreement with experimental findings, particularly for both the C 1s and O 1s core-ionization states of CO, where the relative intensity ratio is less than 0.01 from experiment. Though the deviation increases to about 0.1 in O_2_, DSRG-MRPT3 is considerably better than DSRG-MRPT2 in reproducing these Franck–Condon factors, indicating the importance of third-order corrections in simulating molecules along dissociative pathways.

## 5 Conclusions

This study benchmarks the GAS-DSRG approach for computing core-ionized electronic states using three different molecular benchmark sets. In the GAS-DSRG, core-ionized states are first modeled by a generalized-active-space self-consistent-field reference, followed by a treatment of dynamical electron correlation via the MR-DSRG theory. For small molecules with no more than three non-hydrogen atoms, we applied a state-specific approach, while the state-averaged formalism was essential for modeling the high density of core-ionized states in large organic systems.

The results for the three benchmark sets allow us to draw robust conclusions regarding the accuracy of computed core-ionization energies as a function of the level of truncation of the MR-DSRG treatment. The K-edge ionization energies of first-row elements of small molecules^65^ from the DSRG-MRPT2, DSRG-MRPT3, and MR-LDSRG(2) theories show good agreement with experimental values, with MAEs of less than 0.3 eV and STDs of less than 0.2 eV. These deviations outperform MR-ADC(2)-X^83^ and are comparable to those from EOMIP-CCSDT^65^ and ΔCCSD(T).^66^ Among the three DSRG theories, DSRG-MRPT2 exhibits an MAE of 0.16 eV compared to the experiment, much better than the values of 0.23 eV from DSRG-MRPT3 and 0.26 eV from MR-LDSRG(2). The small difference between DSRG-MRPT3 and MR-LDSRG(2) results suggests that DSRG-MRPT3 is an excellent method for evaluating core-ionized states, balancing accuracy and computational cost. We then evaluated the accuracy of GAS-DSRG theories in assessing core-ionization energies across various sizes of molecules using the CORE65 set.^31^ The DSRG-MRPT3 theory again shows high accuracy in evaluating core-ionization energies across various molecules, exhibiting an overall MAE of only 0.34 eV and a 0.25 eV STD. In comparison, DSRG-MRPT2 shows lower accuracy and consistency due to significant errors in outlier points, primarily attributed to intruder-state problems, highlighting the superiority of DSRG-MRPT3 for assessing core–electron correlations for large molecules. Our GAS-DSRG calculations also reveal intriguing energy order differences compared to GW calculations in core-ionized states of molecules with multiple identical atoms, such as nitrobenzene and phenylacetylene, suggesting that the treatment of core vacancies as localized or delocalized can significantly affect the energies of core-ionized states in XPS simulations. We finally extend our GAS-DSRG into assessing the molecules along the potential energy surface by predicting vibrationally resolved XPS spectra of CO, N_2_, and O_2_.^89−91^ DSRG-MRPT2 and DSRG-MRPT3 theories agree well with experimental spectra with shifts of less than 0.34 eV required for alignment. Although DSRG-MRPT3 shows slightly larger errors than DSRG-MRPT2 in the adiabatic energies, it predicts a more accurate splitting for the ^2^Σ and ^4^Σ states of O_2_^+^. The DSRG-MRPT2/3 vibrational constants are less than 50 cm^–1^ from experimental values and earlier theoretical predictions at the SAC–CI and ADC(4) levels.^89−91^

While the present work shows that the GAS-DSRG method can accurately predict the XPS and TR-XPS of a few core-ionization channels in molecules, it also highlights several challenges associated with multireference excited-state methods. As for core excitations, the GASSCF process remains the most computationally demanding and error-prone component in the GAS-DSRG procedure. Therefore, it is highly desirable to develop an optimized algorithm to generate GASSCF reference states or alternative efficient methods to incorporate core-relaxation effects. Moreover, our work has neglected the evaluation of core-ionization cross-sections due to the complexity of describing continuum states. A practical method for evaluating core-ionization cross-sections at the GAS-DSRG level, such as the Dyson orbitals approach,^122,125^ is needed. Despite these challenges, the GAS-DSRG method is well-suited for simulating the X-ray spectra of transient species during chemical reactions. Examples include time-resolved X-ray photoabsorption spectroscopy of ions^126,127^ or TR-XPS. Further theoretical extensions of the GAS-DSRG combined with nuclear dynamics are currently being developed.

## Data Availability

The data that supports the findings of this study are available within the article and its Supporting Information.

## Supporting Information Available

Active space and number of states used for the core-ionization calculation of the CORE65 set; transition energies obtained using GAS-DSRG for the CORE65 set; and Morse potential parameters and Franck–Condon factors obtained from the GAS-DSRG potential energy surfaces of CO, N_2_, and O_2_ (PDF)
