# Supplementary material for: Benchmark Study of Core-Ionization Energies with the Generalized Active Space-Driven Similarity Renormalization Group
Source: J Chem Theory Comput. 2024 Sep 13;20(18):7990–8000. doi: 10.1021/acs.jctc.4c00835 (PMC11428169; doi:10.1021/acs.jctc.4c00835)
Supplement: Supplementary file 1 — ct4c00835_si_001.pdf [file ct4c00835_si_001.pdf]

Supporting Information: Benchmark Study of Core-Ionization Energies with the Generalized Active Space-Driven Similarity Renormalization Group

Meng Huang and Francesco A. Evangelista*

Department of Chemistry and Cherry Emerson Center for Scientific Computation, Emory University, Atlanta, Georgia, 30322, U.S.A.

E-mail: francesco.evangelista@emory.edu

Table S1: The active space and the number of states averaged for GASSCF-DSRG calculations on the core-ionized state of each molecule. The ground state is calculated with the same active space but one more electron in the GAS1. The elements are labeled by their 1s orbital Hartree-Fock energy if a split-core approximation is applied to perform a GASSCF calculation. (C_1 denotes the calculation uses the lowest carbon 1s orbital in GAS1).

Name	Formula	Active Space	No. of States
methane	CH_4	(1o,1e;8o,8e)	1
ethane	C_2H_6	(2o,3e;11o,10e)	2
ethene	C_2H_4	(2o,3e;12o,12e)	1
ethyne	C_2H_2	(2o,3e;10o,10e)	1
carbon monoxide	CO	(1o,1e;9o,14e) (C/O)	1/1
carbon dioxide	CO_2	(1o,1e;14o,20e) (C)/ (2o,3e;13o,18e) (O)	1/1
tetrafluoromethane	CF_4	(1o,1e;10o,12e) (C)/ (4o,7e;10o,12e) (F)	1/1
fluoromethane	CH_3F	(1o,1e;11o,16e) (C/F)	1/1
trifluoromethane	CHF_3	(1o,1e;8o,8e) (C)/ (3o,5e;8o,8e) (F)	1/3
methanol	CH_3OH	(1o,1e;11o,11e) (C/O)	1/1
formaldehyde	CH_2O	(1o,1e;12o,15e) (C/O)	1/1
dimethyl ether	CH_3OCH_3	(2o,3e;13o,11e) (C)/(1o,1e;11o,9e)(O)	2/1
formic acid	HCOOH	(1o,1e;10o,9e) (C/O)	1/1
acetone	$(CH_3)_2CO$	(2o,3e;5o,4e) ($C_{2/3}$)/ (1o,1e;5o,4e) (C_1/O)	2/1/1
methyl formate	HCO_2CH_3	(1o,1e;8o,4e)	1
acetic acid	CH_3COOH	(1o,1e;8o,4e) (C_1/C_2/O_1/O_2)	1/1/2/1
water	H_2O	(1o,1e;6o,8e)	1
ozone	O_3	(1o,1e;14o,22e) (O_1)/(2o,3e;13o,20e) (O_{23})	1/1
oxygen	O_2	(2o,3e;8o,12e)	1
nitrogen	N_2	(2o,3e;8o,10e)	1
ammonia	NH_3	(1o,1e;7o,8e)	1
hydrogen cyanide	HCN	(1o,1e;10o,13e) (C/N)	1/1
acetonitrile	CH_3CN	(1o,1e;13o,10e) (C_1/C_2/N)	1/1/1
glycine	$C_2H_5NO_2$	(1o,1e;8o,6e) (C_1/C_2/N/O_1/O_2)	1/1/1/1/1
pyridine	C_5H_5N	(1o,1e;6o,6e)	1
pyrrole	C_4H_4NH	(1o,1e;5o,6e)	1
aniline	$C_6H_5NH_2$	(1o,1e;5o,4e)	1
urea	$CO(NH_2)_2$	(1o,1e;6o,6e)(C)/(2o,3e;6o,6e)(N)/(1o,1e;6o,6e)(O)	1/1/1
methylamine	CH_3NH_2	(1o,1e;11o,10e)	1
nitrobenzene	$C_6H_5NO_2$	(6o,11e;8o,10e)(C)/(2o,3e;8o,10e) (N/O)	6/1/1
benzene	C_6H_6	(6o,11e;6o,6e)	2
phenylacetylene	C_8H_6	(8o,15e;8o,8e)	8

Table S2: The core-ionized energies of molecules in the CORE65 data set[1] from experiment and GAS-DSRG theories. Results were calculated with a GASSCF reference and different levels of theory [PT2 = DSRG-MRPT2, PT3 = DSRG-MRPT3] using the cc-pVQZ basis. The energies are in eV.

Name	Formula	Core level	Exp.	PT2	PT3
methane	CH_4	C1s	290.84	290.48	290.53
ethane	C_2H_6	C1s	290.71	290.34	290.45
ethene	C_2H_4	C1s	290.82	290.18	290.69
ethyne	C_2H_2	C1s	291.25	290.69	291.11
carbon monoxide	CO	O1s	542.10	541.83	541.89
carbon monoxide	CO	C1s	296.23	295.73	295.74
carbon dioxide	CO_2	O1s	541.32	540.69	541.00
carbon dioxide	CO_2	C1s	297.70	297.43	297.28
tetrafluoromethane	CF_4	F1s	695.20	694.83	694.94
tetrafluoromethane	CF_4	C1s	301.90	301.70	301.65
fluoromethane	CH_3F	F1s	692.40	692.28	692.16
fluoromethane	CH_3F	C1s	293.56	293.28	293.21
trifluoromethane	CHF_3	F1s	694.10	693.10	694.50
trifluoromethane	CHF_3	C1s	299.16	299.08	298.87
methanol	CH_3OH	O1s	538.88	538.51	538.55
methanol	CH_3OH	C1s	292.30	292.02	292.20
formaldehyde	CH_2O	O1s	539.33	539.06	538.95
formaldehyde	CH_2O	C1s	294.38	294.23	294.17
dimethyl ether	CH_3OCH_3	O1s	538.36	538.21	537.91
dimethyl ether	CH_3OCH_3	C1s	292.17	291.67	291.90
formic acid	HCOOH	O1s (OH)	540.69	540.51	540.21
formic acid	HCOOH	O1s (C=O)	539.02	538.69	538.48
formic acid	HCOOH	C1s	295.75	295.68	295.49
acetone	$(CH_3)_2CO$	O1s	537.73	537.60	537.61
acetone	$(CH_3)_2CO$	C1s (C=O)	293.88	293.49	293.44
acetone	$(CH_3)_2CO$	C1s (CH_3)	291.23	289.38	291.09
methyl formate	HCO_2CH_3	O1s (OCH_3)	539.64	539.57	539.81
methyl formate	HCO_2CH_3	O1s (C=O)	538.24	537.93	538.11
acetic acid	CH_3COOH	O1s (OH)	540.10	540.11	539.46
acetic acid	CH_3COOH	O1s (C=O)	538.31	537.92	538.11
acetic acid	CH_3COOH	C1s (COOH)	295.35	295.41	295.27
acetic acid	CH_3COOH	C1s (CH_3)	291.55	291.24	291.26
water	H_2O	O1s	539.70	539.32	539.26
ozone	O_3	O1s middle	546.44	545.53	545.64
ozone	O_3	O1s terminal	541.75	540.84	541.35
oxygen	O_2	O1s weaker	544.20	543.75	543.78
oxygen	O_2	O1s stronger	543.10	542.72	542.86
nitrogen	N_2	N1s	409.93	409.24	409.46
ammonia	NH_3	N1s	405.52	405.05	405.05
hydrogen cyanide	HCN	N1s	406.80	405.95	405.92

hydrogen cyanide	HCN	C1s	293.50	293.42	293.46
acetonitrile	CH_3CN	N1s	405.58	405.08	405.05
acetonitrile	CH_3CN	C1s (CH_3)	292.88	292.33	292.21
acetonitrile	CH_3CN	C1s (CN)	292.60	292.38	292.29
glycine	$C_2H_5NO_2$	O1s (OH)	540.20	539.93	539.83
glycine	$C_2H_5NO_2$	O1s (C=O)	538.40	538.03	537.78
glycine	$C_2H_5NO_2$	N1s	405.40	405.09	405.15
glycine	$C_2H_5NO_2$	C1s (COOH)	295.20	295.17	295.06
glycine	$C_2H_5NO_2$	C1s (CH_2)	292.30	291.89	291.96
pyridine	C_5H_5N	N1s	404.82	404.27	404.16
pyrrole	C_4H_4NH	N1s	406.18	405.83	405.73
aniline	$C_6H_5NH_2$	N1s	405.31	404.95	404.90
urea	$CO(NH_2)_2$	O1s	537.19	537.17	536.61
urea	$CO(NH_2)_2$	N1s	406.09	405.47	406.05
urea	$CO(NH_2)_2$	C1s	294.84	294.90	294.63
methylamine	CH_3NH_2	N1s	405.17	404.71	404.69
nitrobenzene	$C_6H_5NO_2$	O1s	538.63	536.09	538.03
nitrobenzene	$C_6H_5NO_2$	N1s	411.60	411.03	411.38
nitrobenzene	$C_6H_5NO_2$	C1s (C_1)	292.08	291.17[a]	292.43[a]
nitrobenzene	$C_6H_5NO_2$	C1s (C_{2-4})	291.13	289.67[a]	290.89[a]
benzene	C_6H_6	C1s	290.38	288.85	290.02
phenylacetylene	C_8H_6	C1s (C_3)	290.88	289.62[b]	291.17[c]
phenylacetylene	C_8H_6	C1s (C_2)	290.55	289.13[b]	290.46[c]
phenylacetylene	C_8H_6	C1s (C_{4-6})	290.16	288.82[b]	290.43[c]
phenylacetylene	C_8H_6	C1s (C_1)	289.75	288.81[b]	289.67[c]

[a] The assignments for these transitions are C_4 and C_{1-3} in energy descending order or DSRG-MRPT2/DSRG-MRPT3 results.

[b] The assignments for these transitions are C_3, C_2, C_1 and C_{4-6} in energy descending order for DSRG-MRPT2 results.

[c] The assignments for these transitions are C_2, C_3, C_1 and C_{4-6} in energy descending order for DSRG-MRPT3 results.

Table S3: The vibrational constants calculated for core-ionized states of CO and N_2 . These constants are obtained from fitting the potential energy surface calculated using DSRG-MRPT2/3 levels of theories and cc-pCVQZ-DK basis to Morse potentials. The experimental values, along with the calculated results from CCSD(T)-ADC(4) and SAC-CI theories, are also provided.[2,3] ΔR is the difference in equlibrium bond-length between the core-ionized and ground state. ω_e is the vibrational frequency and $\omega_e\chi_e$ is the anharmonic constant. The calculated relative Franck-Condon factors, I, with respect to the $\nu = 0$ value are also reported.

Parameters	DSRG-MPRT2	DSRG-MPRT3	Exp.	ADC(4)	SAC-CI
CO^+, C1s ($^2\Sigma_g$)					
ΔR (Å)	−0.0563	−0.0571	−0.0514	−0.0440	−0.0510
ω_e (cm^{-1})	2464	2506	2479	2437	2444
$\omega_e\chi_e$ (cm^{-1})	22	21	23		25
$I(\nu = 1)/I(\nu = 0)$	0.594	0.634	0.640	0.521	0.626
$I(\nu = 2)/I(\nu = 0)$	0.139	0.164	0.161	0.101	0.133
$I(\nu = 3)/I(\nu = 0)$	0.016	0.021	0.019	0.009	0.012
CO^+, O1s ($^2\Sigma_g$)					
ΔR (Å)	0.0395	0.0321	0.0370		0.0280
ω_e (cm^{-1})	1824	1914	1864.0		
$\omega_e\chi_e$ (cm^{-1})	16	15	7		9
$I(\nu = 1)/I(\nu = 0)$	0.341	0.256	0.248		0.016
$I(\nu = 2)/I(\nu = 0)$	0.065	0.038	0.030		0.015
${N_2}^+$, N1s ($^2\Sigma_g$)					
ΔR (Å)	−0.0188	−0.0237	−0.0186	−0.0174	−0.0230
ω_e (cm^{-1})	2388	2457	2414	2440	2486
$\omega_e\chi_e$ (cm^{-1})	21	20			
$I(\nu = 1)/I(\nu = 0)$	0.140	0.084			
${N_2}^+$, N1s ($^2\Sigma_u$)					
ΔR (Å)	−0.0235	−0.0283	−0.0240	−0.0210	−0.0270
ω_e (cm^{-1})	2387	2452	2407	2437	2492
$\omega_e\chi_e$ (cm^{-1})	21	20			
$I(\nu = 1)/I(\nu = 0)$	0.095	0.049			

Table S4: The vibrational constants calculated for core-ionized states of O_2. These constants are obtained from fitting the potential energy surface calculated using DSRG-MRPT2/3 levels of theories and cc-pCVQZ-DK basis to Morse potentials. The experimental values are also listed, with the u and g states with the same spin multiplicity unresolved.[4] ΔR is the difference in equlibrium bond-length between the core-ionized and ground state. ω_e is the vibrational frequency and $\omega_e\chi_e$ is the anharmonic constant. The calculated relative Franck-Condon factors, I, with respect to the $\nu = 0$ value are also reported.

	DSRG-MPRT2	DSRG-MPRT3	Exp.
O_2^+, O1s ($^2\Sigma_g$)			
ΔR (Å)	0.0738	0.0599	
ω_e (cm^{-1})	1107	1184	1130
$\omega_e\chi_e$ (cm^{-1})	20	19	
$I(\nu=1)/I(\nu=0)$	0.7505	0.4585	0.5411
$I(\nu=2)/I(\nu=0)$	0.3135	0.1214	0.1692
$I(\nu=3)/I(\nu=0)$	0.0983	0.0245	0.0406
$I(\nu=4)/I(\nu=0)$	0.0261	0.0042	0.0840
$I(\nu=5)/I(\nu=0)$	0.0063	0.0007	0.0160
O_2^+, O1s ($^2\Sigma_u$)			
ΔR (Å)	0.075	0.062	
ω_e (cm^{-1})	1101	1178	
$\omega_e\chi_e$ (cm^{-1})	20	19	
$I(\nu=1)/I(\nu=0)$	0.7589	0.4557	
$I(\nu=2)/I(\nu=0)$	0.3221	0.1226	
$I(\nu=3)/I(\nu=0)$	0.1018	0.0257	
$I(\nu=4)/I(\nu=0)$	0.0269	0.0046	
$I(\nu=5)/I(\nu=0)$	0.0063	0.0007	
O_2^+, O1s ($^4\Sigma_g$)			
ΔR (Å)	0.0221	0.0154	
ω_e (cm^{-1})	1403	1482	1450
$\omega_e\chi_e$ (cm^{-1})	18	17	
$I(\nu=1)/I(\nu=0)$	0.0645	0.0177	0.0482
$I(\nu=2)/I(\nu=0)$	0.0010	0.0000	0.0010
O_2^+, O1s ($^4\Sigma_u$)			
ΔR (Å)	0.0738	0.0599	
ω_e (cm^{-1})	1107	1184	
$\omega_e\chi_e$ (cm^{-1})	20	19	
$I(\nu=1)/I(\nu=0)$	0.0547	0.0136	
$I(\nu=2)/I(\nu=0)$	0.0004	0.0000	

References

(1) Golze, D.; Keller, L.; Rinke, P. Accurate Absolute and Relative Core-Level Binding Energies from GW. *J. Phys. Chem. Lett.* **2020**, *11*, 1840–1847.

(2) Matsumoto, M.; Ueda, K.; Kukk, E.; Yoshida, H.; Tanaka, T.; Kitajima, M.; Tanaka, H.; Tamenori, Y.; Kuramoto, K.; Ehara, M.; Nakatsuji, H. Vibrationally resolved C and O 1s photoelectron spectra of carbon monoxides. *Chem. Phys. Lett.* **2006**, *417*, 89–93.

(3) Ueda, K.; Püttner, R.; Cherepkov, N. A.; Gel'mukhanov, F.; Ehara, M. High resolution X-ray photoelectron spectroscopy on nitrogen molecules. *Eur. Phys. J. Special Topics* **2009**, *169*, 95–107.

(4) Sorensen, S. L.; Børve, K. J.; Feifel, R.; de Fanis, A.; Ueda, K. The O 1s photoelectron spectrum of molecular oxygen revisited. *J. Phys. B: At. Mol. Opt. Phys.* **2008**, *41*, 095101.
